# Papillary renal cell carcinoma: current and controversial issues

**DOI:** 10.1097/MOU.0000000000001000

**Published:** 2022-06-09

**Authors:** Silvia Angori, João Lobo, Holger Moch

**Affiliations:** a Department of Pathology and Molecular Pathology, University Hospital Zurich, Zurich, Switzerland; b Department of Pathology, Portuguese Oncology Institute of Porto (IPOP); c Cancer Biology and Epigenetics Group, IPO Porto Research Center (GEBC CI-IPOP), Portuguese Oncology Institute of Porto (IPO Porto) & Porto Comprehensive Cancer Center (P.CCC), R. Dr António Bernardino de Almeida; d Department of Pathology and Molecular Immunology, ICBAS–School of Medicine and Biomedical Sciences, University of Porto (ICBAS-UP), Porto, Portugal; e Faculty of Medicine, University of Zurich, Zurich, Switzerland

**Keywords:** emerging entities, histopathology, molecular pathology, papillary renal cell carcinoma, renal tumours

## Abstract

Papillary renal cell carcinoma (pRCC) is the second most frequent renal cancer subtype and represents 15–20% of all RCC. Classification of pRCC is changing because novel tumour entities have been discovered in the last years. In this review, we summarise recent studies relevant for the understanding of the molecular complexity and the broader differential diagnosis of pRCC.

**Recent findings:**

It has been 25 years ago, that pRCC was morphologically subdivided into type 1 and type 2. Recently described tumour entities in the 2022 WHO classification challenged this concept and allow a new view on the molecular background in pRCC. Biphasic hyalinizing psammomatous RCC and papillary renal neoplasm with reversed polarity are emerging tumour entities derived from the new concept of molecularly defined RCC subtypes. Immune checkpoint inhibition and tyrosine kinase inhibitors have been introduced as the new backbone in the first-line treatment of advanced pRCCs. To identify novel targeted treatments for patients with pRCC it is crucial to investigate the specific molecular background of pRCC considering emerging pRCC subtypes.

**Summary:**

In the future, a deeper understanding of the correlation between molecular aberrations and new pRCC subtypes may improve the classification of pRCC patients and could reveal potential predictive biomarkers for each subgroup.

## INTRODUCTION – EPIDEMIOLOGY, AND HISTOPATHOLOGY OF PAPILLARY RENAL CANCER

Although renal cell carcinoma (RCC) represents only 2% of cancer diagnoses and deaths, its incidence has more than duplicated in developed regions in the past decades [1] with more than 76,000 new cases and 14,000 deaths recorded in 2020 in the United States [2]. Incidence and mortality rates are higher in developed countries due to the presence of modifiable environmental exposures like smoking and diet, in addition to the contribution of nonmodifiable factors like male gender and ethnicity [3,4]. Survival is highly dependent on disease stage, being higher (>90%) in the overall 65% of patients presenting with localised disease, many diagnosed incidentally on imaging [4]. However, a clinically relevant proportion of patients (16%) are diagnosed with distant metastatic disease, which has a dismal prognosis (13.9% 5-year relative survival). There is an unmet need of novel targeted therapies for these metastatic patients, especially since RCC is generally chemo- and radio-resistant [5].

The biggest challenge to this has been the tremendous heterogeneity of renal cancers [6]. RCCs are grouped on several categories by the most recent World Health Organization (WHO) Classification [7] (which will be updated in 2022). Around 75% of RCCs correspond to clear cell RCCs (ccRCCs), which have been the most studied in the field of biomarkers and targeted therapies, leaving nonccRCC patients with less treatment options [8].

Papillary RCC (pRCC) comprises the second most frequent RCC subtype (10–15% of RCCs). Although advocated to have a better prognosis in direct comparison to ccRCC in organ-confined stage, it is reported that pRCC histology in disseminated disease displays poorer outcomes compared to ccRCC [9]. pRCC is, however, a very heterogeneous disease. The first attempt to subclassify pRCC based on morphology was proposed in 1997 by Delahunt and Eble, who divided tumours in type 1 pRCC (slender papillae, with a single layer of small basophilic or amphophilic cuboidal cells lining them) and type 2 pRCC (broader papillae, filled with several layers of large eosinophilic cells, with nuclear pseudostratification and larger nucleoli) [10]. This was later supported by molecular data and adopted by the WHO classification [11], and corroborated by large studies, such as The Cancer Genome Atlas (TCGA) analyses [12–14]. Although some clinical studies suggest type 2 pRCC to have higher nuclear grade and other histopathological features of aggressiveness [15,16], this did not translate to worse patient outcome when adjusted for stage and other variables [17,18]. Moreover, it is now recognised that mixtures of type 1 and 2 areas frequently coexist [19], and it is possible that these represent a progression of low to high tumour grade [20^▪^]. Finally, the spectrum of pRCC (particularly of type 2 pRCC) has suffered several modifications over the past years [20^▪^]. 

**Box 1:**
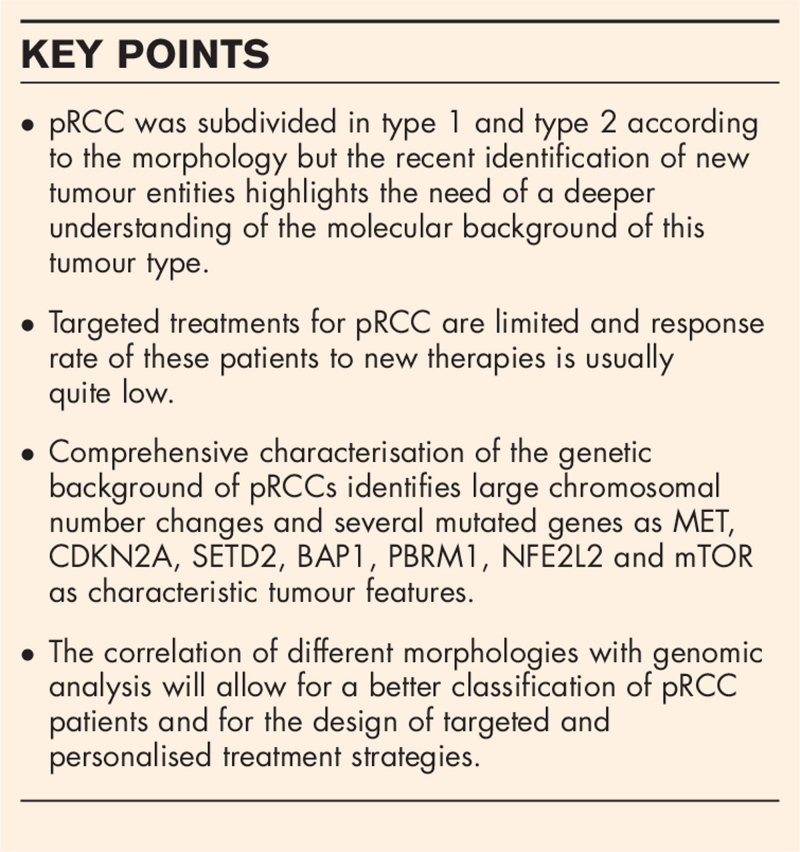
no caption available

## THE SPECTRUM OF RENAL CANCER SUBTYPES WITH PAPILLARY GROWTH – RECENT AND EMERGING ENTITIES

Various works revisiting series of RCCs with papillary features and studying their molecular and clinical background allowed for discrimination of new tumour entities [21^▪▪^,22^▪^,^23^,^24^], already incorporated in the WHO classification (e.g. tubulocystic RCC [25], clear cell papillary RCC [26], fumarate-hydratase deficient RCC [27] and *TFE3* or *TFEB*-translocated RCC [28]). Additionally, some emerging entities have been proposed [29,30], for which more data is currently being gathered, further purifying the true pRCC family of tumours and decreasing the proportion of cases diagnosed as ’unclassified RCC’ [31,32].

Further, current evidence does not support the discrimination of so-called ’oncocytic pRCC’ as an independent entity, since oncocytic change maybe be observed in otherwise typical type 1 or 2 pRCC [33] or in papillary renal neoplasm with reversed polarity (see below). Indeed, a subset of cancers previously considered as type 2 or ’oncocytic pRCC’ are now known to correspond to fumarate hydratase-deficient RCCs [34]. Although their morphology can be quite variable, some features can suggest the diagnosis, such as the large reddish inclusion-like nucleoli surrounded by a clear halo [35]. The distinction is important since these are aggressive cancers, with frequent dissemination, and can point to the discovery of a yet undiagnosed hereditary and leiomyomatosis and renal-cell cancer syndrome (HLRCC) syndrome due to germline mutations in *FH*[36], which can also have implications for treatment [37,38].

Other tumour entities which could have been considered in past series in the pRCC family of tumours include MiT translocated RCCs, both *TFE3*-translocated RCC (Xp11 translocation) and *TFEB*-translocated RCC (t(6;11)) [39]. The variety of architectural patterns mixed together within one tumour, with papillary, nested and tubular growth and presence of clear and eosinophilic cells (biphasic appearance) can suggest the diagnosis [40], which is confirmed by fluorescence *in situ* hybridisation (FISH) [41,42]. More recently, *TFEB*-amplified RCCs were also described, which have high-grade cytological features and aggressive behaviour [22^▪^,^43^]. *ALK*-translocated RCC has also been reported and considered as a very rare RCC subtype [44]. It has the widest range of morphologies described, including papillary features. Its discrimination from pRCC or nonclassified RCC by FISH testing is relevant since current *ALK* inhibitors have demonstrated clinical efficacy [45,46].

Our knowledge on the morphology and molecular background of pRCC has continued to evolve, and currently a set of emerging entities is proposed. Although their prevalence is overall low [20^▪^], continuing studying and accumulating evidence on these entities may contribute to better assessment of patients’ prognosis and treatment decisions.

Biphasic squamous-alveolar RCC, the most frequent of these entities [20^▪^], shows a phenotype closer to type 1 pRCC. The tumour is composed of two cell populations, one of large eosinophilic cells, with higher nuclear grade and squamoid features clustered together in nests and surrounded by a second population of smaller cells, with lower nuclear grade, giving the lower-power impression of multiple alveolar/organoid structures [47]. The large cell population is evidenced by specifically staining for cyclinD1 and CD57 [48,49]. Emperipolesis is another characteristic feature, seen in all cases of a large series [50], considered by others to be more precisely cytophagocytosis of neutrophils [51]. Frequent transition to otherwise classical papillary areas, different proportions of the alveolar pattern between tumours and the typical gains of chromosomes 7/17 in all cases support that these tumours are within the pRCC (type 1) morphological spectrum [50,52]. Data supports that *MET* is a major driver of these RCCs in particular, and the high frequency of *MET* mutation should trigger investigations of therapy with *MET* inhibitors [53].

Biphasic hyalinizing psammomatous RCC is another emerging entity with evident papillary/tubulo-papillary architecture. It also shows admixture of two cell populations, one of small cells, sometimes spindle-shaped, arranged predominantly around basement membrane material, giving the impression of pseudo-rosettes (a feature also observed in *TFEB*-translocated RCC) or forming branching nodules or clusters within larger acini and tubules which are formed by larger eosinophilic cells. The mixture of the two gives the low-power impression of a glomerular pattern [54]. The stroma is sclerotic and frequently shows psammoma bodies. Although EMA stains preferentially the small cells, CK7 stains predominantly the large cell population. This tumour phenotype is associated with somatic *NF2* mutations. This entity was recently described in a single study of eight patients (one of which died from disease), and therefore needs additional work to clarify about its clinical relevance and biology, namely if such *NF2* mutations are the true driver genetic abnormalities. Recently, *NF2* gene inactivation was described in a cohort of advanced stage pRCC, which could have implications for risk stratification [55] and for targeted therapies, as mentioned above.

Another recently described emerging entity is papillary renal neoplasm with reversed polarity [56]. Tumours are usually small and can be partly cystic [57]. They have a quite characteristic morphology, recapitulated in several recent publications [56,58–63], composed of thin papillae with hyalinised cores filled with a single row of eosinophilic cells with small uniform nuclei (sometimes with optical clearing) aligned and pushed against the apical pole (reversed polarity), frequently with peritumoral lymphoid aggregation. In the past, they have probably been fit into a ’low-grade oncocytic pRCC’ designation, due to their low-grade cytology and absence of aggressive features, in line with their good clinical behaviour [61]. Although classical gains of chromosomes 7 and 17 are found, GATA3 and L1CAM nuclear positivity and *KRAS* mutations are particular findings that can aid in diagnosis.

**FIGURE 1:**
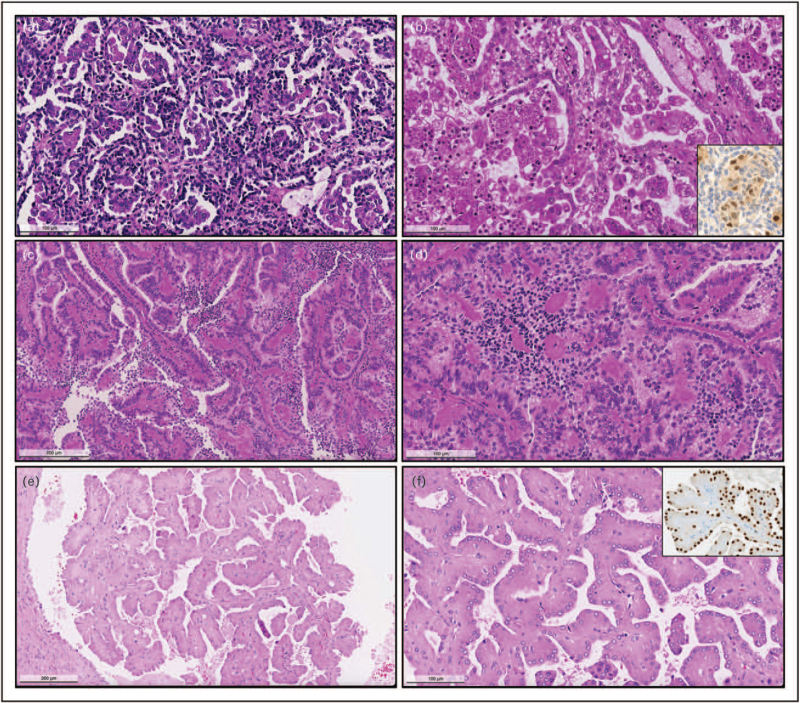
Histopathological features of emerging entities within the spectrum of papillary renal cell cancer. a,b: Biphasic squamous-alveolar renal cell carcinoma. The tumour is composed of two cell populations, one of smaller cells with low nuclear grade surrounding a second population of larger eosinophilic cells, with higher nuclear grade, forming alveolar structures (a). The larger cells have squamoid cytoplasmic features, and frequently phagocyte neutrophils. Scattered foamy histiocytes typically found in papillary renal cell carcinomas are seen (b). The large cells stain for cyclinD1 (inset). c,d: Biphasic hyalinizing psammomatous renal cell carcinoma. The tumour is also composed of two cell populations, one of small cells with dark nuclei, sometimes spindle-shaped, and other of larger cells lining papillae (c). The small cells tend to surround strongly eosinophilic basement membrane material, forming pseudo-rosettes (d). e,f: Papillary renal neoplasm with reversed polarity. The tumour is small, and is formed within a cystic space (e). The papillae are hyalinized and tumour cells are strongly eosinophilic. The nuclei are small, low grade, show optical clearing and are pushed against the apical pole of the cells (f). The nuclei stain with GATA3 (inset).

Additional emerging entities/disease phenotypes which need further investigation include Warthin-like pRCC [64], a papillary tumour with eosinophilic papillae and with stroma filled by brisk lymphocytic infiltrate, resembling Warthin tumour of the parotid gland, and also thyroid-like follicular RCC [65,66], which resembles thyroid follicular architecture, with cuboidal cells around colloid material, possibly having papillary foci [67]. The latter has recently been reported to harbour *EWSR1*-*PATZ1* fusions and also to possibly have aggressive behaviour [68–70], and more studies are required to fully understand the clinical relevance of this phenotype. Illustrative photomicrographs of emerging entities are presented in Fig. 1.

## MOLECULAR BACKGROUND OF PAPILLARY RENAL CANCER – RECENT DEVELOPMENTS AND TREATMENT OPPORTUNITIES OF METASTATIC PAPILLARY RENAL CELL CARCINOMA

The novelties in classification together with a deeper investigation of the molecular background of pRCCs may also open the way for specific targeted therapies for these patients. Among different molecular features, large somatic copy number changes of pRCC have been reported decades ago by G. Kovacs *et al.*[71,72,73^▪^]. Chromosome 7 and 17 gains are nearly universally seen in type 1 pRCC, whereas type 2 pRCC is more characterised by gains in chromosomes 12, 16 and 20. Other chromosomal gains are also described for chromosome 2 and 3 [72,74,75]. Interestingly, TCGA identifies three subgroups of pRCC with different copy number alteration pattern. Low-grade tumours compose one subgroup predominantly with gain of chromosomes 7, 12, 16, 17 and 20. The other two subgroups behave completely differently. Although one group shows only few alterations, the other one is characterised by high genome instability and loss of chromosome 9p associated with poor survival [14,76]. In few cases, intra-chromosomal rearrangements of chromosomes 1, 2 and 3 are also described [72].

Recently, several genes have been found to be mutated in pRCC. Germline or somatic mutations in the proto-oncogene *MET* are found mainly in type 1 pRCC. Mutations or promoter hypermethylation of *CDKN2A* are strongly associated with aggressive pRCC. Loss of *CDKN2A*, encoding for p16, results in increased expression of cell-cycle related genes [77]. In addition, mutations in chromosome modifier genes *SETD2, BAP1*, and *PBRM1* are also described in pRCC. Although these genes, commonly mutated also in ccRCC, are associated with loss of chromosome 3, no chromosomal change is usually observed in pRCC tumours. Other common mutated genes are *FAT1, FLCN, TERT, NF2, NFE2L2, STAG2* and *TP53*, which are involved in chromatin modification, cell cycle and metabolism [14,78,79]. Numerous mutated genes in pRCC are components of well-described cancer pathways like Hippo, mTOR or p53 [14]. The Hippo pathway controls cell proliferation by inhibiting the transcriptional co-activator protein YAP1. In pRCC, loss of NF2 leads to over-activation of YAP1 and, consequently, to abnormal cellular growth [80,81].

Aberrations in genes as *mTOR*, *PIK3CA*, *PTEN* or *FBXW7*, *RB1*, and *TP53* state the involvement of mTOR and p53 pathways in pRCC. These two pathways are crucial for cell division and proliferation, apoptosis and response to stress [82,83]. In cancer cells, aberrations in these pathways can contribute to tumorigenesis and progression of RCCs [84–86]. Mutations in *TP53* correlate, indeed, with poor patient survival [87]. Lastly, mRNA enrichment analysis on TCGA data shows over-activation of the Nrf2-ARE pathway in aggressive pRCCs [14]. Mutations in key genes of this pathway such as *NFE2L2, CUL3* or *Keap1* lead to the constitutive activation of Nrf2, a transcription factor responsible for cell proliferation under oxidative stress conditions [88,89].

Over-expression of the Cerebellar degeneration-related protein 2 (Cdr2) was also described as a potential biomarker in pRCC due to the reduction of HIF response under hypoxia [90].

*FH* is often mutated in the aggressive HLRCC [14], which represents an own and independent tumour subtype. Since *FH* mutations are also seen in sporadic tumours, this tumour entity will be renamed into *FH*-deficient RCC in the up-coming WHO classification.

A summary of treatment options for advanced/metastatic pRCC patients is presented in Table 1. Targeted treatments for pRCC are limited and new therapies have not significantly improved patient survival [91]. However, the heterogeneous molecular background of pRCC tumour should be taken into consideration for personalised targeted strategies. For example, pRCC shows recurrent alteration in *MET* by gene amplification or mutations. MET is a tyrosine kinase transmembrane receptor that binds the Hepatocyte Growth Factor (HGF) to regulate cell growth, proliferation and angiogenesis. Aberrant activation of MET leads to tumorigenesis, metastases migration and invasiveness [92]. Given the frequent MET over-expression in pRCC, several inhibitors have been used in the treatment of these patients. Cabozantinib, a dual MET-VEGF inhibitor, was approved in 2016 for the treatment of pRCC patients and it became a broad treatment for all nonccRCCs [91,93]. Recent studies showed that Savolinib, a potent and selective MET kinase inhibitor, has higher efficacy compared to Sunitinib in the treatment of tumours with MET alterations [94,95^▪▪^]. MET activation is also often associated with the Epidermal Growth Factor Receptor (EGFR) expression in pRCC. Therefore, combination of MET and EGFR inhibitors (Tivantinib and Erlotinib [96–98]) can represent a treatment strategy in MET-driven pRCC tumours [97,99].

**Table 1:** Treatment options for advanced/metastatic pRCC patients

	Drug	Class	Target
First-line treatment	Savolitinib [94]	TKI	MET
	Pembrolizumab [105^▪^]	ICI	PD-1 receptor
	Cabozantinib [93]	TKI	MET, RET, AXL, VEGFR2, FLT3, c-KIT
	Sunitinib [110]	TKI	PDGR, VEGF-R, CD117
Second-line treatment	Everolimus [111]	TKI	mTOR
	Tivozanib [98]	TKI	VEGF-1, VEGF-2, VEGF-3, c-kit, PDGR
Other treatment	Tivantinib [96]	TKI	c-MET
	Erlotinib [97]	TKI	EGFR

pRCC, papillary renal cell carcinoma. First- and second-line treatment options for pRCC patients based on the latest European Society for Medical Oncology guidelines [95^▪▪^]. For each compound, the class of action (Tyrosine Kinase Inhibitors or Immune checkpoint inhibitors) and the drug targets are shown.

According to the latest European Society for Medical Oncology guidelines, systematic treatments of advanced or metastatic nonccRCC patients include immune checkpoint inhibitors (ICIs) such as anti-PD1/PDL1 antibodies and tyrosine kinase inhibitors (TKIs) [95^▪▪^]. Although PD1-PDL1 inhibitors can restore the T-cell response against tumour cells [100–102], TKIs are able to target the downstream targets of the Von Hippel-Lindau pathway such as vascular endothelial growth factor (VEGF) or platelet-derived growth factor (PDGF). These drugs are designed on the molecular consequences of the inactivation of the tumour suppressor gene *Von Hippel-Lindau*, the most common alteration on ccRCC but less frequent in pRCC. Therefore, treatment of nonccRCCs with these compounds showed reduced effectiveness and low response rate [103].

The efficacy of the PD1 inhibitor, Pembrolizumab, was tested for the first time in 2021 on 165 nonccRCCs in a phase II clinical trial (ClinicalTrials.gov identifier: NCT02853344). The results showed promising clinical activity in the overall nonccRCC population: in particular, better overall response was detected in pRCC patients compared to chRCC, the third major RCC subtype. The clinical responses correlate with high PD-L1 expression [104,105^▪^]. According to these results, Pembrolizumab was introduced as a first-line treatment option for pRCC. Nivolumab alone or in combination with the VEGF inhibitor, Cabozantinib, also showed promising efficacy in pRCC [106–108]. Alternative first or second-line treatment options for nonccRCCs also include mTOR-targeted therapies such as Cabozantinib, Sunitinib, Pazopanib and Everolimus [109–111]. Importantly, nonccRCC showed a worse response to these compounds and shorter survival compared to advanced ccRCC. Robust evidence is missing for third-line treatment strategies [95^▪▪^,^99^]. These findings suggest that specific molecular characteristics in different subsets of pRCC can determine different targeting approaches.

## CONCLUSION

To conclude, continuing studying the diverse morphologies of pRCC and respective molecular alterations, and establishing important genotype-phenotype correlations will contribute to a better risk stratification of patients with pRCC, enabling the discovery of prognostic biomarkers. This will lead to more targeted and personalised treatment strategies for these nonccRCC renal cancer patients.

## Acknowledgements

*None.*

*Author contributions: Literature review: S.A. and J.L. Drafting of the paper: S.A. and J.L. Supervision and final editing: H.M. All authors read and approved the final paper.*

### Financial support and sponsorship

*H.M. receives a Swiss National Science Foundation grant (No. S-87701-03-01).*

### Conflicts of interest

*There are no conflicts of interest.*

## REFERENCES AND RECOMMENDED READING

Papers of particular interest, published within the annual period of review, have been highlighted as:

▪ of special interest
▪▪ of outstanding interest
